# Deletion of P2X7 Receptor Decreases Basal Glutathione Level by Changing Glutamate-Glutamine Cycle and Neutral Amino Acid Transporters

**DOI:** 10.3390/cells9040995

**Published:** 2020-04-16

**Authors:** Hana Park, Ji-Eun Kim

**Affiliations:** Department of Anatomy and Neurobiology, Institute of Epilepsy Research, College of Medicine, Hallym University, Chuncheon 200-702, Korea; M19050@hallym.ac.kr

**Keywords:** cysteine, GCLC, glutamate cysteine ligase, GSH synthetase, GSH, GSS, NAC, SIN-1

## Abstract

Glutathione (GSH) is an endogenous tripeptide antioxidant that consists of glutamate-cysteine-glycine. GSH content is limited by the availability of glutamate and cysteine. Furthermore, glutamine is involved in the regulation of GSH synthesis via the glutamate–glutamine cycle. P2X7 receptor (P2X7R) is one of the cation-permeable ATP ligand-gated ion channels, which is involved in neuronal excitability, neuroinflammation and astroglial functions. In addition, P2X7R activation decreases glutamate uptake and glutamine synthase (GS) expression/activity. In the present study, we found that P2X7R deletion decreased the basal GSH level without altering GSH synthetic enzyme expressions in the mouse hippocampus. P2X7R deletion also increased expressions of GS and ASCT2 (a glutamine:cysteine exchanger), but diminished the efficacy of N-acetylcysteine (NAC, a GSH precursor) in the GSH level. SIN-1 (500 μM, a generator nitric oxide, superoxide and peroxynitrite), which facilitates the cystine–cysteine shuttle mediated by xCT (a glutamate/cystein:cystine/NAC antiporter), did not affect basal GSH concentration in WT and P2X7R knockout (KO) mice. However, SIN-1 effectively reduced the efficacy of NAC in GSH synthesis in WT mice, but not in P2X7R KO mice. Therefore, our findings indicate that P2X7R may be involved in the maintenance of basal GSH levels by regulating the glutamate–glutamine cycle and neutral amino acid transports under physiological conditions, which may be the defense mechanism against oxidative stress during P2X7R activation.

## 1. Introduction

Glutathione (GSH) is an endogenous tripeptide antioxidant that consists of glutamate-cysteine-glycine. Glutamate cysteine ligase (GCLC) converts glutamate and cysteine (mostly derived from cystine, the oxidized dimer form of cysteine) to the dipeptide γ-glutamylcysteine (γGluCys), which is the rate-limiting step in cellular GSH synthesis. Thereafter, GSH synthetase (GSS) generates GSH by adding glycine (derived from exogenous glycine or serine) to γGluCys in an ATP-driven reaction [1]. In the brain, astrocytes play an important role in GSH metabolism. Astrocytes uptake glutamate and convert it into glutamine via glutamine synthase (GS), which is transferred to neurons to serve as a precursor for glutamate synthesis [2]. In addition, astrocytes provide neighboring neurons with the GSH precursors [3]. Thus, the glutathione content is limited by the availability of glutamate and cysteine in astrocytes, but cysteine is the rate-limiting precursor of GSH synthesis in neurons [4]. Furthermore, glutamine concentration affects GSH synthesis rate [5], since glutamine is used for GSH synthesis via the glutamate–glutamine cycle mediated by glutaminase (GLS) [1,4,6,7].

The solute carrier 1 (SLC1) family includes neutral amino acid exchange proteins that preferentially transfer the substrates **a**lanine, **s**erine and **c**ysteine (termed ASC). Among them, SLC1A4 and SLC1A5 are known as ASCT1 and ASCT2, respectively. Although ASCT1 and ASCT2 have high affinity for these amino acids, ASCT2 differs from ASCT1 by also accepting glutamine and asparagine as high affinity substrates [8,9]. In addition, a cystine/glutamate transporter (xCT or SLC7a11) exchanges cystine for glutamate (or cysteine) with a molar ratio of 1:1 by the substrate gradients across the plasma membrane under physiological conditions [10,11]. Thus, these membrane transporters also support GSH synthesis to supply neutral amino acids.

The P2X7 receptor (P2X7R) is one of the cation-permeable ATP ligand-gated ion channels which is involved in neuronal excitability, neuroinflammation and astroglial functions [12,13,14,15,16,17,18]. Since P2X7R activation accelerates free radical generations [15,16], it is plausible that the defense mechanism against P2X7R-mediated oxidative stress may be present to maintain the GSH level during P2X7R activation under physiological conditions. Interestingly, P2X7R activation decreases glutamate uptake and GS activity in astrocytes, although P2X7R cannot affect the release of GSH. Furthermore, P2X7R activation regulates glutamate- and ASCT2-mediated d-serine release from astrocytes [17,18,19,20,21]. Thus, it is likely that P2X7R may be involved in the glutamate–glutamine cycle and neutral amino acid transports, which affect GSH levels [1,4,6,7], but is still unveiled.

Here, we demonstrate that P2X7R deletion decreases the basal GSH level in a mouse hippocampus, although it did not influence GCLC, GSS and GLS expression levels. However, P2X7R deletion increased GS and ASCT2 expressions without altering xCT expression. Furthermore, P2X7R deletion prevents the diminished efficacy of *N*-acetylcysteine (NAC, a GSH precursor) in GSH synthesis induced by SIN-1. Thus, these findings suggest that P2X7R may modulate GSH levels by regulating the glutamate–glutamine cycle and neutral amino acid transports via ASCT2 and xCT under physiological conditions.

## 2. Materials and Methods

### 2.1. Experimental Animals and Chemicals

We used male C57BL/6J (P2X7R*^+/+^*, wild type, WT) and P2X7R^−/−^ (knockout, KO) mice (60 to 90 days old, 25–30 g, The Jackson Laboratory, USA) in the present study. Animals were given a commercial diet and water ad libitum under controlled conditions (22 ± 2 °C, 55% ± 5% humidity, and a 12-h light/12-h dark cycle). All experimental protocols described below were approved by the Institutional Animal Care and Use Committee of Hallym University (Chuncheon, South Korea, Hallym 2018-3, 30th April 2018). Every effort was made to reduce the number of animals employed and to minimize animal discomfort. All reagents were obtained from Sigma-Aldrich (St. Louis, MO, USA), except as noted.

### 2.2. NAC Treatment and Acute Brain Slices

NAC acts as a GSH precursor and a free radical scavenger per se [22]. When NAC is directly applied in the bath during acute brain slice culture with SIN-1 (a generator of nitric oxide, superoxide and peroxynitrite [22], see below), SIN-1 might react with NAC and subsequently reduce the efficacy of NAC in GSH synthesis. Thus, we pretreated NAC in vivo to avoid the action of NAC as a free radical scavenger.

Five hours after NAC (70 mg/kg, i.p.) or vehicle treatment, animals were sacrificed by cervical dislocation and decapitated. Brains were rapidly removed and placed in ice-cold cutting solution (composition in mM: KCl 3, NaH_2_PO_4_ 1.25, MgSO_4_ 6, NaHCO_3_ 26, CaCl_2_ 0.2, glucose 10 and sucrose 220). Coronal sections (300 μm thickness) were cut on a vibratome (Campden Instruments Limited, Loughborough, UK) and slices were subsequently transferred to oxygenated ACSF (composition in mM: NaCl 124, KCl 2.5, NaHCO_3_ 26, KH_2_PO_4_ 1.25, MgSO_4_ 2, CaCl_2_ 2.5, glucose 10 and sucrose 4, pH 7.4, bubbled with 95% O_2_ and 5% CO_2_) at room temperature [23]. Cutting solution was 300–305 mOsm/L. After warming to 34 °C for 30 min, the ACSF was exchanged again, and slices were then held at room temperature. Individual slices were then transferred to a chamber and perfused with oxygenated ACSF at 2 mL/min [23]. After 10 min of incubation, SIN-1 (500 μM) or vehicle was added to each chamber for 2 h. After culture, slices were used for immunohistochemistry or GSH assay.

### 2.3. GSH Assay

Brain slices were sonicated with 0.5 mL of 5% sulfosalicylic acid and centrifuged at 10,000× *g* for 10 min at 4 °C. The supernatant was mixed with 1 mm dithiobis-2-nitrobenzoic acid and 1 mm EDTA in 100 mm sodium phosphate buffer, pH 7.5, and 1 mm NADPH and 200 U/mL of glutathione reductase were added [24]. GSH standards were treated identically, and optical absorbance of samples and standards was measured at 405 nm. Values were normalized to protein content as determined with a BCA protein assay kit (Thermo Scientific) [25].

### 2.4. Immunohisto Chemistry

Brain slices were immersed into 4% paraformaldehyde in 0.1 M PB (pH 7.4) overnight. The brain tissues were cryoprotected by infiltration with 30% sucrose overnight. Thereafter, the slices were frozen and sectioned with a cryostat at 30 μm. Free-floating sections were washed 3 times in PBS (0.1 M, pH 7.3) and incubated with 3% bovine serum albumin in PBS for 30 min at room temperature. Later, sections were incubated with glial fibrillary acidic protein (GFAP, a marker for astrocytes) or a cocktail solution containing MAP1 and 4-HNE antisera (Table 1) in PBS containing 0.3% Triton X-100 overnight at room temperature. Thereafter, sections were visualized with appropriate Cy2- and Cy3-conjugated secondary antibodies. Immunoreaction was observed using an Axio Scope microscope (Carl Zeiss Korea, Seoul, South Korea). To establish the specificity of the immunostaining, a negative control test was carried out with preimmune serum instead of the primary antibody. All experimental procedures in this study were performed under the same conditions and in parallel. To measure fluorescent intensity, 5 areas/animals (300 μm^2^/area) were randomly selected within the hippocampus (5 sections from each animal, *n* = 7 in each group). Thereafter, mean fluorescence intensity of 4-HNE signals on each section was measured by using AxioVision Rel. 4.8 software. Intensity measurements were represented as the number of a 256 gray scale. The intensity of each section was standardized by setting the threshold level (mean background intensity obtained from five image inputs). Manipulation of the images was restricted to threshold and brightness adjustments to the whole image.

### 2.5. Western Blot

Animals were decapitated under urethane anesthesia (1.5 g/kg, i.p.). Animal protocols were approved by the Institutional Animal Care and Use Committee of Hallym University (Chuncheon, Korea). The hippocampus was rapidly dissected out and homogenized in lysis buffer. The protein concentration in the supernatant was determined using a Micro BCA Protein Assay Kit (Pierce Chemical, Dallas, TX, USA). Thereafter, Western blot was performed by the standard protocol (*n* = 7 in each group). The primary antibodies used in the present study are listed in Table 1. The bands were detected and quantified on an ImageQuant LAS4000 system (GE Healthcare Korea, Seoul, South Korea). As an internal reference, rabbit anti-β-actin primary antibody (1:5000) was used. The values of each sample were normalized with the corresponding amount of β-actin. 

### 2.6. Data Analysis

All data obtained from the quantitative measurements were analyzed using Student’s *t*-test and one-way ANOVA to determine statistical significance. Bonferroni’s test was used for post hoc comparisons. A *p*-value below 0.05 was considered statistically significant.

## 3. Results

### 3.1. P2X7R Deletion Increases GS and ASCT2 Expression

**Figure 1:**
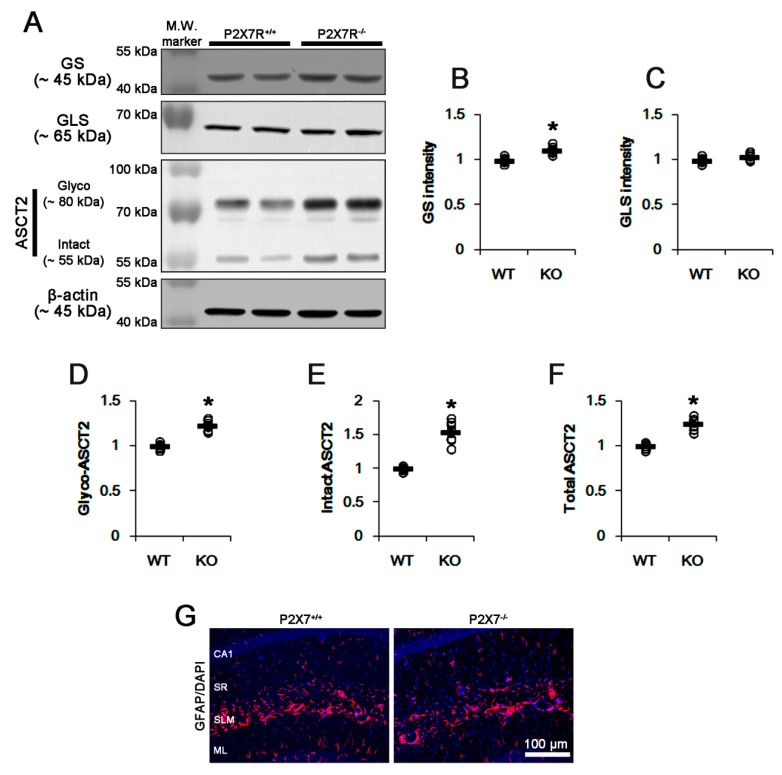
Effects of P2X7R deletion on expressions of glutamine synthase (GS), glutaminase (GLS), a glutamine:cysteine exchanger (ASCT2) and glial fibrillary acidic protein (GFAP). P2X7R deletion increases GS and ASCT2, but not GLS, expressions. (**A**) Representative Western blots of GS, GLS and ASCT2 expressions. (**B**–**F**) Quantification of GS (**B**), GLS (**C**), glycosylated ASCT2 (Glycol-ASCT2, **D**), intact ASCT2 (**E**,**F**) total ASCT2 levels based on Western blot data. Open circles indicate each individual value. Horizontal bars indicate mean value (mean ± S.E.M.; * *p* < 0.05 vs. WT animals; n = 7, respectively). (**G**) Representative photos for GFAP expression in the hippocampus. P2X7R deletion does not result in reactive astrogliosis in the hippocampus. Abbreviations: CA1, CA1 pyramidal cell layer; SR, stratum radiatum; SLM, stratum lacunosum-moleculare; ML, molecular layer of the dentate gyrus.

Since P2X7R activation decreases glutamate uptake and GS activity/expression in vitro [17], we investigated the effect of P2X7R deletion on GS expression in vivo. GS expression in P2X7R KO mice was slightly, but significantly, higher than that in WT mice (*p* < 0.05 vs. WT animals; n = 7; Figure 1A,B and Supplementary Figure S1). However, P2X7R KO mice showed no difference in GLS expression, as compared to WT mice (Figure 1A,C and Supplementary Figure S1). These findings indicate that P2X7R deletion may increase GS activity/expression more than GLS, which would increase glutamine concentration. The increased GS expression and glutamine concentration potentially facilitates glutamine efflux from astrocytes by inducing ASCT2 trafficking [26,27]. Thus, we confirmed whether the upregulation of GS expression induced by P2X7R deletion affects ASCT2 expression. Consistent with a previous study [28,29], the present study showed two ASCT2 bands: a *N*-linked glycosylated band (70~90 kDa) and an intact band (non-glycosylated, ~55 kDa) (Figure 1A and Supplementary Figure S1). P2X7R deletion elevated *N*-linked glycosylated-ASCT2 levels approximately 1.23-fold of WT level (*p* < 0.05 vs. WT animals; *n* = 7; Figure 1A,D and Supplementary Figure S1). P2X7R deletion also increased intact ASCT2 and total ASCT2 levels to approximately 1.55- and 1.25-fold of WT level (*p* < 0.05 vs. WT animals; n = 7; Figure 1A,E,F and Supplementary Figure S1). P2X7R deletion did not lead to reactive astrogliosis in the hippocampus (Figure 1G). Since *N*-glycosylation of ASCT2 at N163 and N212 sites is critical for trafficking to membrane [30], our findings indicate that P2X7R deletion may increase glutamine concentration and ASCT2-mediated glutamine efflux without inducing reactive astrogliosis.

### 3.2. P2X7R Deletion Reduces GSH Concentration

**Figure 2:**
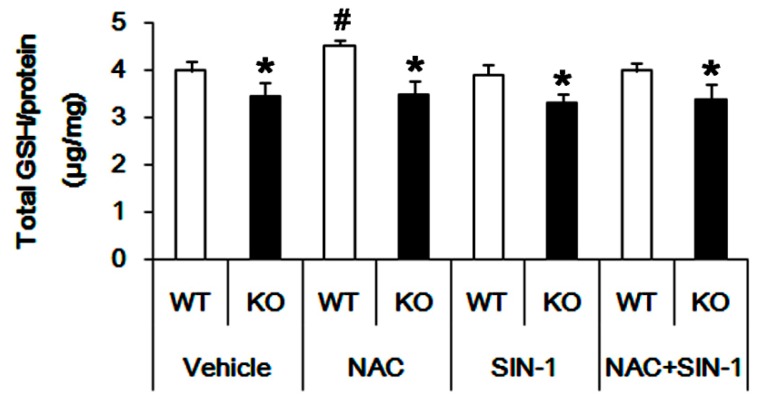
GSH assay in the hippocampus. P2X7R deletion decreases basal GSH level. NAC increases GSH level in WT animals but not P2X7R KO mice in vivo. Although SIN-1 does not affect GSH level in the hippocampal slices of both animals, it reduces GSH concentration to a basal level in NAC-treated WT animals but not P2X7R KO mice, ex vivo (mean ± S.E.M.; *,^#^ *p* < 0.05 vs. WT animals and vehicle-treated animals, respectively; n = 7, respectively).

**Figure 3:**
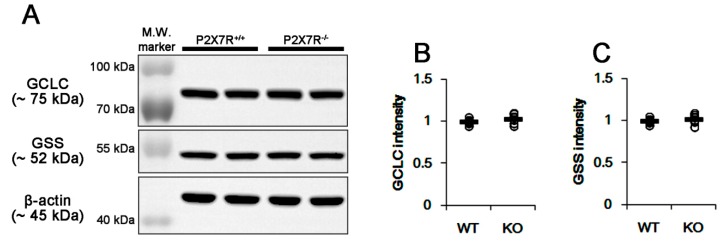
Effects of P2X7R deletion on expression of GSH synthetic enzymes. P2X7R deletion does not affect GCLC and GSS expressions. (**A**) Representative Western blots of GCLC and GSS expressions. (**B**,**C**) Quantification of GCLC (**B**) and GSS (**C**) levels based on Western blot data. Open circles indicate each individual value. Horizontal bars indicate mean value (mean ± S.E.M.; n = 7, respectively).

ASCT2-mediated glutamine release from astrocytes is required for alanine, serine or cysteine in extracellular space [27]. Considering glutamine and cysteine as GSH precursors [1,4,6,7], it is likely that upregulated ASCT2 expression would elevate GSH concentration in P2X7R KO mice via facilitation of glutamine-cysteine antiport as well as the increased GS activity. Thus, we measured GSH concentration in the hippocampus. Unexpectedly, we found that total GSH level in P2X7R KO mice (3.45 ± 0.29 μg/mg protein; *p* < 0.05 vs. WT animals; n = 7; Figure 2) was lower than that in WT mice (3.98 ± 0.19 μg/mg protein; Figure 2). Thus, we explored if P2X7R deletion would influence GSH synthetic enzyme expressions. However, P2X7R deletion did not affect GCLC and GSS expressions (Figure 3A–C and Supplementary Figure S2.).

Next, we applied NAC (a GSH precursor, 70 mg/kg, i.p.) to validate whether P2X7R deletion affects the efficacy of NAC in GSH synthesis. In WT mice, NAC elevated GSH concentration to 4.53 ± 0.09 μg/mg protein (*p* < 0.05 vs. vehicle; n = 7; Figure 2). However, NAC did not affect GSH concentration in P2X7R KO mice (3.49 ± 0.27 μg/mg protein, Figure 2). These findings indicate that P2X7R deletion may decrease basal GSH levels by reducing the yield of GSH precursor transports.

### 3.3. P2X7R Deletion Inhibits xCT-Mediated NAC Transport

**Figure 4:**
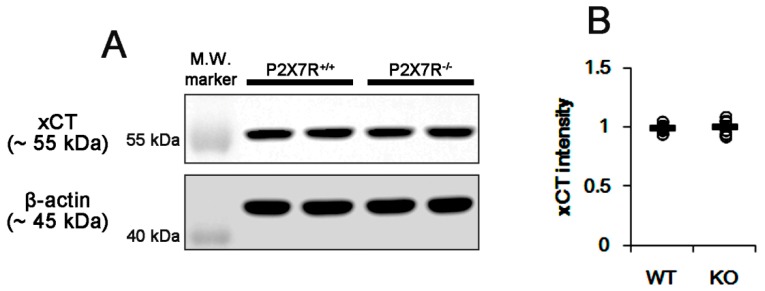
Effects of P2X7R deletion on xCT expression. P2X7R deletion does not affect xCT expression. (**A**) Representative Western blots of xCT expression. (**B**) Quantification of xCT level based on Western blot data. Open circles indicate each individual value. Horizontal bars indicate mean value (mean ± S.E.M.; * *p* < 0.05 vs. WT animals; n = 7, respectively).

Since xCT is one of the transporters for cystine and NAC into the intracellular space [10,11,31,32,33], we investigated whether P2X7R deletion affects xCT expression. However, there was no difference in xCT expression between WT and P2X7R KO mice (Figure 4A,B). These findings indicate that P2X7R deletion may decrease basal GSH levels by inhibiting xCT-mediated cystine or NAC transport without affecting xCT expression. To confirm this, we applied SIN-1 (500 μM) in acute brain slice culture, since this SIN-1 concentration facilitates the xCT-mediated cystine–cysteine shuttle via the increased cysteine release from cells [34].

**Figure 5:**
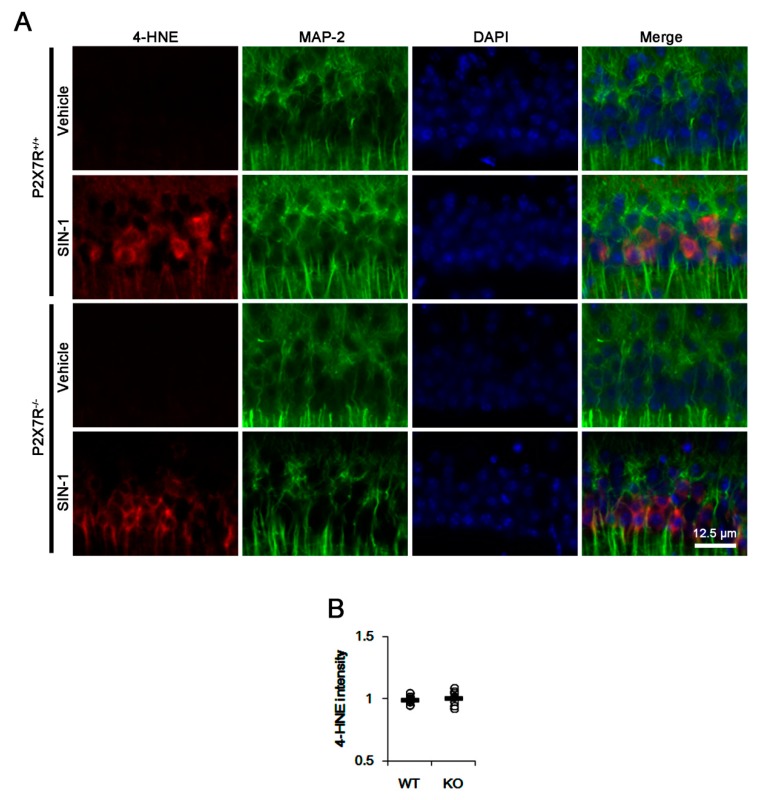
Effects of P2X7R deletion on 4-HNE synthesis induced by SIN-1. SIN-1 induces 4-HNE signals in the CA1 regions. P2X7R deletion does not affect 4-HNE signals after SIN-1 treatment. (**A**) Representative images of 4-HNE induction. (**B**) Quantification of 4-HNE fluorescent intensity. Open circles indicate each individual value. Horizontal bars indicate mean value (mean ± S.E.M.; n = 7, respectively).

As compared to the vehicle, SIN-1 similarly increased 4-hydroxy-2-nonenal (4-HNE) signals in both WT and P2X7R KO mice (Figure 5A,B). Since SIN-1 generates nitric oxide, superoxide, nitric oxide and peroxynitrite, which produces 4-HNE as a stable end production of lipid peroxidation [22,35], our findings indicate that P2X7R deletion may not influence SIN-1-induced oxidative stress. Furthermore, SIN-1 did not affect basal GSH concentration in both WT (3.91 ± 0.21 μg/mg protein) and P2X7R KO (3.31 ± 0.18 μg/mg protein) mice (Figure 2). However, SIN-1 effectively reduced the efficacy of NAC in GSH synthesis (3.99 ± 0.17 μg/mg protein) in WT mice (*p* < 0.05 vs. NAC; n = 7; Figure 2), but not in P2X7R KO mice (3.37 ± 0.32 μg/mg protein; Figure 2). These findings indicate that P2X7R deletion may inhibit xCT function, which would decrease the cystine or NAC transport, and that P2X7R knockout may elevate ASCT2 expression as an adaptive response to exchange glutamine for cysteine.

## 4. Discussion

**Figure 6:**
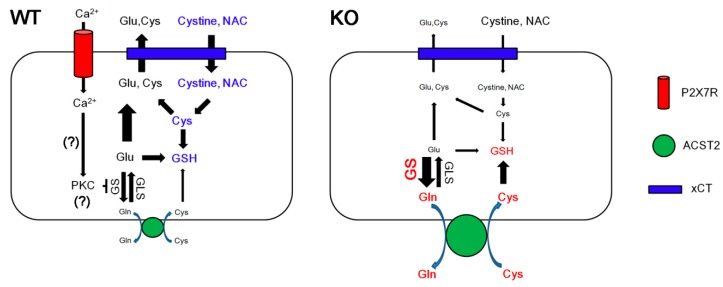
Scheme of roles of P2X7R in basal GSH levels based on the present data and a previous report [17]. Under physiological conditions, P2X7R activation increases Ca^2+^ influx, which would inhibit GS expression by PKC activation without altering GLS expression. This mode of regulation maintains the yield of the glutamate (Glu)–glutamine (Gln) cycle and xCT-mediated uptake of cysteine (Cys) precursors such as cystine/NAC, which increases GSH level. However, P2X7R deletion leads to the upregulation of GS expression, which inhibits xCT activity due to reduced glutamate level. Instead, the elevated glutamine level accelerates ACST2-mediated glutamine–cysteine exchanges. The reductions in both glutamate and cystine levels diminish the GSH level.

The major findings in the present study are that P2X7R deletion reduced the basal GSH level, accompanied by increased GS and ACST2 expressions. In addition, the lack of P2X7R prevented the reduced efficacy of NAC in GSH synthesis induced by SIN-1, suggesting the relevance between P2X7R and xCT system (Figure 6).

Glutamate is one of the excitatory neurotransmitters and substrates for GSH as well as bioenergetics. In astrocytes, the up-taken glutamate is converted to glutamine via GS [2,3]. Interestingly, P2X7R activation inhibits GS expression/activity in protein kinase C (PKC)-dependent manner [17]. Consistent with this report, the present study shows that P2X7R deletion increases GS expression without changing GLS. Furthermore, P2X7R knockout enhanced the *N-*glycosylated ASCT2 expression that is an indicative of its cell surface expression [30]. Since the upregulation of GS expression exerts ASCT2 trafficking into the astroglial surface [26], our findings indicate that P2X7R deletion may increase GS-mediated glutamine synthesis in astrocytes, and subsequently facilitate ASCT2-mediated glutamine efflux from astrocytes [27].

On the other hand, glutamine is involved in GSH synthesis via the glutamate–glutamine cycle, mediated by GLS [4,6,7]. Considering the roles of ASCT2 as a glutamine:cysteine exchanger [4,6,7,8,9], it is plausible that P2X7R deletion may elevate basal GSH concentration. Unexpectedly, the present study showed that P2X7R knockout resulted in the reverse phenomenon. Thus, it is simply interpreted that the lower GSH level in P2X7R KO mice would be a consequence of the facilitating GSH efflux or inhibiting GSH synthesis. However, P2X7R is not involved in GSH efflux from astrocytes [20], and P2X7R deletion did not affect GLS, GCLS and GSS expressions in the present study. Therefore, our findings suggest that P2X7R deletion may decrease GSH concentration due to the reduced turnover of glutamine to glutamate by GS overexpression or the excessive ASCT2-mediated glutamine efflux. Furthermore, it is likely that P2X7R deletion may reduce the demand of GSH for maintenance of the intracellular redox state, since P2X7R activation generates reactive oxygen species (ROS) via p38 mitogen-activated protein kinase (p38 MAPK) and c-Jun N-terminal kinase (JNK) [36]. Indeed, intervention of P2X7R signaling hinders production of nitric oxide, peroxynitrite and hydroxyl radicals [15,16,37,38,39]. In addition, the GSH level affects P2X7R expression and its activity [40]. Thus, our findings indicate that P2X7R may facilitate GSH synthesis by regulating the glutamate–glutamine cycle and cystine uptake to prevent free radical damage during its activation under physiological conditions.

In the present study, we found that P2X7R deletion abrogated the GSH-increasing capacity of NAC without altering xCT expression. Since the GSH conversion from NAC is required for subphysiological glutamine level [5], it is likely that the increased glutamine concentration by the upregulated GS expression in P2X7R KO mice may diminish the efficacy of NAC in GSH synthesis. In addition, xCT plays a role as a NAC transporter [31,32,33], although NAC is a membrane-permeable cysteine precursor that does not require active transport [41]. Under physiological conditions, xCT exchanges cysteine for glutamate with a molar ratio of 1:1, which is driven by the substrate gradients across the plasma membrane [10,11,31,32,33,42]. xCT also constitutes a cystine –cysteine shuttle whereby cystine uptake drives cysteine release [34]. Since the xCT-mediated cystine transport is involved in the supply of cysteine for GSH synthesis [43], it is also plausible that P2X7R deletion would diminish xCT-mediated NAC transport without affecting xCT expression, due to the reduced glutamate–glutamine cycle by GS overexpression. Interestingly, the present study shows that SIN-1 (500 μM) effectively decreased the efficacy of NAC in GSH synthesis in WT mice, but not in P2X7R KO mice, although SIN-1 did not affect basal GSH levels in both mice. GSH does not affect the decomposition kinetics of SIN-1 [44]. In addition, this concentration of SIN-1 affects GSH level by glutamate-inhibitable cystine uptake and an increased rate of cysteine release from cells without changing total GSH concentration [34,45,46]. Indeed, 4-HNE production induced by SIN-1 was unaffected by the distinct endogenous GSH level between WT and P2X7R KO mice, indicating that 500 μM SIN-1 may not evoke GSH degradation or consumption. Therefore, our findings suggest that P2X7R deletion may inhibit xCT functions, and upregulate ASCT2 expression as an adaptive response for xCT inhibition under physiological conditions.

There is no experimental evidence or literature concerning P2X7R interactions with xCT. However, over 50 different proteins have been identified to physically interact with P2X7R, since P2X7R contains a long intracellular C-terminus that constitutes 40% of the whole protein [47]. In particular, P2X7R activation results in translocation of the Ca^2+^-dependent PKC isoforms, such as PKCα and PKCβI, but not the Ca^2+^-independent isoform PKCδ in osteoclasts [48]. However, P2X7R activated PKCδ and PKCμ in rat parotid acinar salivary cells and astrocytes [49,50]. Furthermore, P2X7R interacts with PKCγ in astrocytes [51]. In the present study, we speculate that P2X7R-mediated PKC activation may regulate GS expression, since P2X7R activation inhibits GS expression/activity in PKC-dependent manner [17]. Indeed, the P2X7R-mediated decreases in GS activity/expression are restored by GF109203X (an inhibitor of Ca^2+^-dependent PKCα, PKCβI, PKCβII, and PKCγ) and Gö6979 (an inhibitor of Ca^2+^-dependent PKCα and PKCβI) [17]. In addition, stimulation of PKCδ (Ca^2+^-independent, diacylglycerol-dependent PKC isoform) in glial cells also causes a marked decrease in the expression of GS [52]. With respect to these previous reports, PKCα, PKCβ, PKCγ and PKCδ are involved in P2X7R-mediated GS regulation. Further studies are needed to elucidate the PKC isoform specificity of P2X7R-mediated GS regulation.

Recently, the decrease of GSH level has been shown to induce cognitive decline and neuronal death during aging and neurodegenerative diseases [53,54]. Interestingly, P2X7R is a therapeutic target in the treatment of epilepsy [12,13,14,55]. Indeed, benzoylbenzoyl-ATP (BzATP, an P2X7R agonist) increases GSH concentration in the cerebrum through penicillin-induced epileptiform activity, which is reversed by A-438079, a P2X7R antagonist [56]. In contrast, Brilliant Blue G (another P2X7R antagonist) attenuated the decreased GSH level in the cortex of a pentylenetetrazol-induced kindling epilepsy model [57]. Therefore, it is worth further investigating the relevance between P2X7R and GSH in neurodegenerative diseases, the aging process and epilepsy.

## 5. Conclusions

The present study demonstrates for the first time the P2X7R-mediated regulation of GSH levels in the hippocampus. Under physiological conditions, P2X7R deletion reduced the basal GSH level and the efficacy of NAC in GSH synthesis by modulating GS and ASCT2 expression, and presumably by xCT inhibition. In addition, P2X7R deletion prevented the decrease in the efficacy of NAC in GSH production induced by SIN-1. Therefore, we suggest that P2X7R may be involved in the regulation of GSH metabolism under physiological conditions.

## Figures and Tables

**Table 1:** Primary antibodies used in the present study.

Antigen	Host	Manufacturer (Catalog Number)	Dilution Used
4-HNE	Rabbit	Alpha Diagnostic (# HNE11-S)	1:1000 (IH)
ASCT2	Rabbit	Alomone labs (#ANT-082)	1:500 (WB)
GCLC	Rabbit	Abcam (#ab190685)	1:2000 (WB)
GFAP	Mouse	Millipore (#MAB3402)	1:1000 (IH)
GLS	Rabbit	Abcam (#ab93434)	1:1000 (WB)
GS	Mouse	Millipore (#MAB302)	1:1000 (WB)
GSS	Rabbit	Abcam (#ab133592)	1:2000 (WB)
MAP2	Mouse	Millipore (#MAB3418)	1:100 (IH)
xCT	Rabbit	Abcam (#ab175186)	1:1000 (WB)
β-actin	Mouse	Sigma (#A5316)	1:5000 (WB)

IH: Immunohistochemistry; WB: Western blot.

## Supplementary Materials

The following are available online at https://www.mdpi.com/2073-4409/9/4/995/s1, Figure S1: Full-length gel images of western blot data in Figure 1A, Figure S2: Full-length gel images of western blot data in Figure 3A, Figure S3: Full-length gel images of western blot data in Figure 4A.
