# Supplementary material for: Deletion of P2X7 Receptor Decreases Basal Glutathione Level by Changing Glutamate-Glutamine Cycle and Neutral Amino Acid Transporters
Source: Cells. 2020 Apr 16;9(4):995. doi: 10.3390/cells9040995 (PMC7226967; doi:10.3390/cells9040995)
Supplement: Supplementary file 1 [file cells-09-00995-s001.pdf]

Supplementary Information

Deletion of P2X7 Receptor Decreases Basal Glutathione Level by Changing Glutamate-Glutamine Cycle and Neutral Amino Acid Transporters

Hana Park and Ji-Eun Kim *

Department of Anatomy and Neurobiology, Institute of Epilepsy Research, College of Medicine, Hallym University, Chuncheon 200-702, Korea; M19050@hallym.ac.kr

* Correspondence: jieunkim@hallym.ac.kr; Tel.: +82-33-248-2522; Fax: +82-33-248-2525

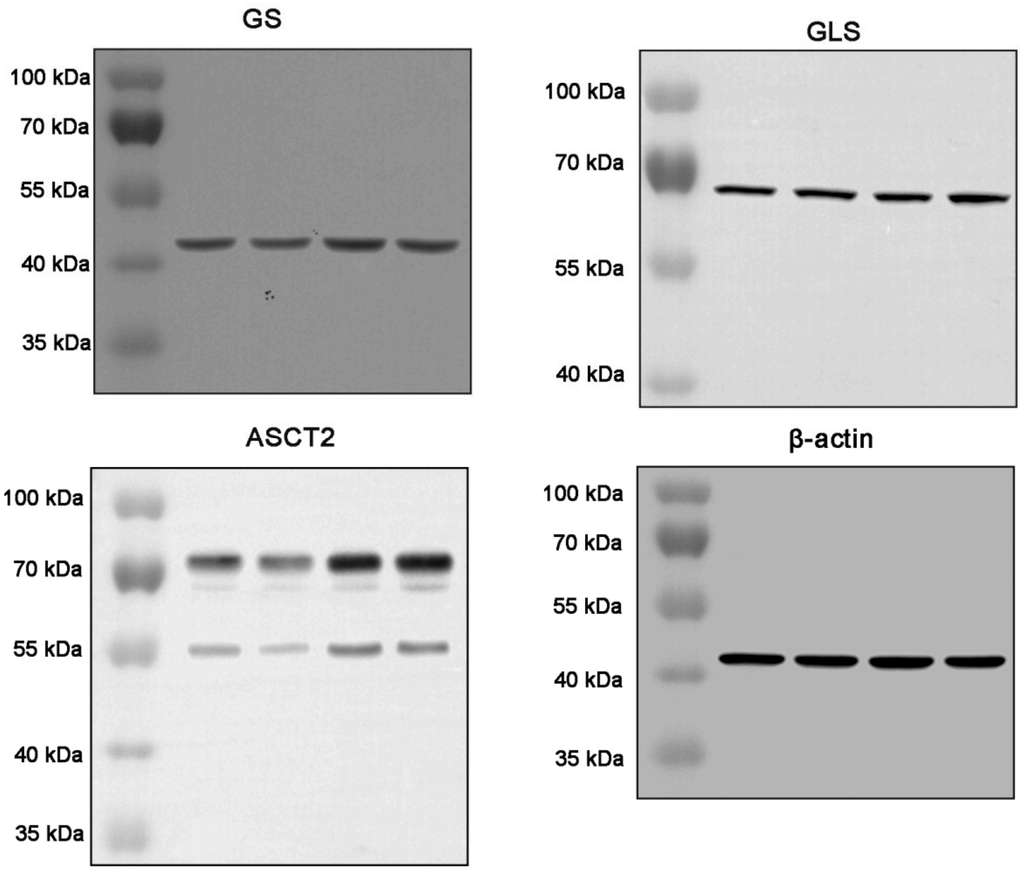

Supplementary Figure S1. Full-length gel images of Western blot data in Figure 1A.

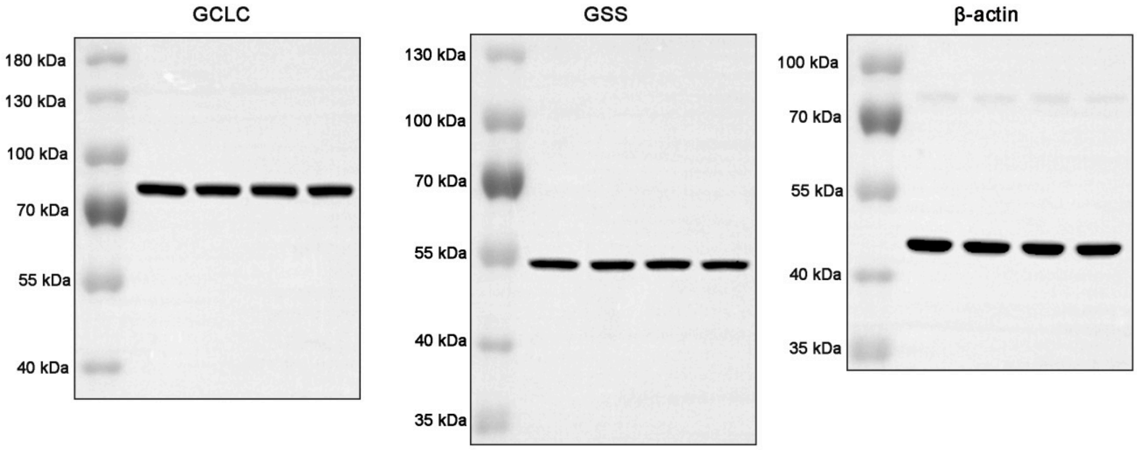

Supplementary Figure S2. Full-length gel images of Western blot data in Figure 3A.

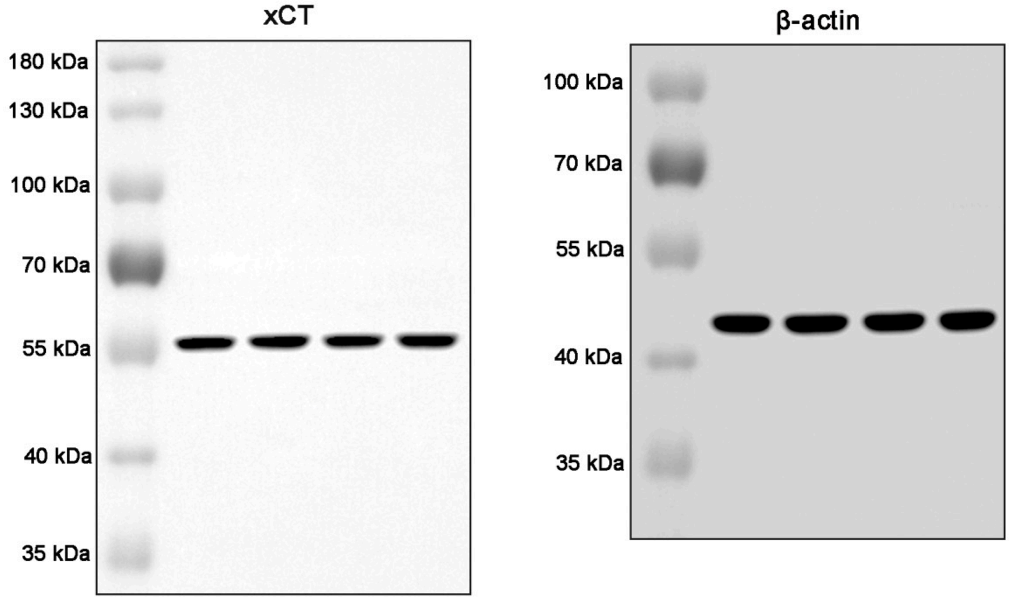

Supplementary Figure S3. Full-length gel images of Western blot data in Figure 4A.
